# Hardware optimization for effective switching power reduction during data compression in GOLOMB rice coding

**DOI:** 10.1371/journal.pone.0308796

**Published:** 2024-09-26

**Authors:** R. Sakthivel, Ch. Vijayalakshmi, M. Vanitha, Kareem M. AboRas, Waleed Mohammed Abdelfattah, Yazeed Yasin Ghadi, Ch. Rami Reddy

**Affiliations:** 1 School of Electronics Engineering, Vellore Institute of Technology, Vellore, India; 2 Department of Electronics and Communication Engineering, Vignan Institute of Technology and Science, Hyderabad, India; 3 School of Computer Science Engineering and Information Systems, Vellore Institute of Technology, Vellore, India; 4 Faculty of Engineering, Department of Electrical Power and Machines, Alexandria University, Alexandria, Egypt; 5 General Subject Department Department, University of Business and Technology, Jeddah, Saudi Arabia; 6 Department of Computer Science and Software Engineering, Al Ain University, Abu Dhabi, UAE; 7 Applied Science Research Center, Applied Science Private University, Amman, Jordan; 8 Department of Electrical and Electronics Engineering, Joginpally B R Engineering College, Hyderabad, India; Chandigarh University, INDIA

## Abstract

Loss-less data compression becomes the need of the hour for effective data compression and computation in VLSI test vector generation and testing in addition to hardware AI/ML computations. Golomb code is one of the effective technique for lossless data compression and it becomes valid only when the divisor can be expressed as power of two. This work aims to increase compression ratio by further encoding the unary part of the Golomb Rice (GR) code so as to decrease the amount of bits used, it mainly focuses on optimizing the hardware for encoding side. The algorithm was developed and coded in Verilog and simulated using Modelsim. This code was then synthesised in Cadence Encounter RTL Synthesiser. The modifications carried out show around 6% to 19% reduction in bits used for a linearly distributed data set. Worst-case delays have been reduced by 3% to 8%. Area reduction varies from 22% to 36% for different methods. Simulation for Power consumption shows nearly 7% reduction in switching power. This ideally suggest the usage of Golomb Rice coding technique for test vector compression and data computation for multiple data types, which should ideally have a geometrical distribution.

## 1. Introduction

Data compression refers to use of any particular technique that reduces the number of bits used to represent the same amount of data. This is done using a variety of techniques that have been vastly explored in recent decades. Often, it can be found that the type of compression technique used, usually pertains to the specific application where the said compression technique is intended for. Golomb Rice coding has been used in data compression for a long time. Image compression seems to be its earliest uses [1]. This technique has been prevalently used for image compression in medical field [2]. This particular compression technique has been fairly popular among researchers working with ECG data.Electrocardiogram (ECG) data is a recording of electrical motion of a living heart over a period of time which is produced using electrodes placed on the patient’s body. Different ECG compression techniques like TURNING POINT, AZTEC, CORTES, FFT and DCT have been implemented and studied [3–5]. Several other research works have focussed on Dictionary based ECG data compression [6]. Golomb Rice coding has also been used for ECG data compression using asynchronous time encoding analog to digital converter [7]. While several other researchers have used adaptive linear prediction with adaptive GR coding to improve results [8]. There have been researches in wavelet based methods for ECG compression and their VLSI implementation [9]. Researchers Nicky H. Bellani & Payal Ghutke have used GR codes to implement their own test vector compression in VLSI [10]. Several others have implemented GR encoding methods for test data compression [11–14]. Beamforming algorithm architectures are used for medical ultrasound imaging [15,16]. A 15:4 Approximate Compressor based multiplier is used for image processing [16–22]. Low power low complexity based lossless data compression were proposed and discussed in [18,19,23,24]. IC design algorithm used in the backend require data compression for thermal aware computing and for test data response compaction as discussed in [20,25].

### 1.1 Motivation and background

The demand for lossless audio data compression is the need of the hour for applications like high-quality sound on commercial media, studio music processing, advertising materials and in the production of radio and television programs, films (post-production). Though there compression standards like MP3, MP4 they are more computational intensive so a need for less computation with less power consumption and more efficient lossless data compression for geometric data distribution is satisfied by the choice of Golomb codes [26].

The world of signal processing and multimedia demands a high quality compression as the requirement of bandwidth increases on daily basis with simultaneous requirement of high quality compression [27].

Retrieval of digital information plays a major role in Digital Libraries, Search Engines etc. The biggest challenge is the number of users and the volume of data in the website. This demands the index construction, index compression, query processing, and ranking algorithm, which will be better handled by Golomb code technique [28].

With the advancement in SoC design the gate count had increased multiple fold and test vector required for testing the SoC had also increased multiple times, this naturally increase the cost, area, testing power and testing time of an IC so test vector compression and test response compaction became a viable solution. The compression algorithms like Golomb code technique plays an inevitable role in VLSI Testing [29,30].

## 2. Literature survey

There are huge volumes of works which have reported on image and signal compression but there are very few works which are being reported on the hardware development for the compression algorithms for test data compression, ECG signal compression etc.

In addition there are works reported on sensitive date lossless compression. Dominik Rzepka [21] discuss about the lossless data compression for an ECG signal using selective linear prediction methodology and it proves to be more effective for the asymmetrical numerical systems. It also well suit for the multichannel signal.

Hang Wang et al. [16] developed an efficient compression based hardware for reducing the on chip memory area requirement by proposing a line buffer architecture. The proposed structure for the compression algorithm provides a good signal to noise ratio. It increases the throughput with reasonable reduction in the hardware cost.

Seongmoon Wang et al. [22] improves the test data compression ratio to a great extent and thereby increases the fault coverage for the Circuit under Test (CUT) in the Automatic Test Engine (ATE). This works aims for compression in test vectors and its response generated in the Linear Feedback shift Register (LFSR) and the ATE respectively.

Xiaoke Qin et al. [23] worked on bitstream compression technique. Bitstream compression is important in reconfigurable system design since it reduces the bitstream size and the memory requirement. It also improves the communication bandwidth and thereby decreases the reconfiguration time.Wei Jhih Wang et al. [24] worked on modified version of dictionary-based code compression. Memory is a key factor in embedded system design. Code compression is a technique used in embedded systems to reduce the memory usage. Bit Mask-based code compression is applied in this work to increase the compression ratio without increase in the hardware cost. HarisLekatsas et al. [25] presents a suitable algorithm that will combine approximate compression techniques with bit-toggling reduction and it explores the various tradeoffs. We take advantage of the approximations introduced to modify codes and reduce bit-toggling, while maintaining the compression performance and decoding speed.

### 2.1. Existing golomb rice coding technique

The Golomb-Rice (GR) encoding technique is a part of a larger family of prefix codes formed by S.W. Golomb around 1966 as an alternative to the Huffman coding [31]. Golomb Rice coding takes its name from S.W. Golomb and R. F. Rice. Rice described his own modification of the original GR encoding technique where the divisors are a power of two[32].GR codes are optimally suited for encoding symbols from a data set where the probability distribution is exponential (for some parameters of the exponential distribution). However, for a finite alphabet GR codes are neither optimal nor complete.

The GR family of codes is characterised by an important parameter ‘M’. This parameter is a non-negative integer. In order to encode any input (non-negative integers), the encoded output is framed in two parts namely unary and different code. First, the unary part is calculated and then the different part is calculated. These two parts are then concatenated to form a single line of code, which is then known as GR code. Table 1 illustrate the GR encoding scheme for M = 4. Table 1 illustrate the GR encoding scheme for M = 4.

**Table 1:** GR encoding example for M = 4 [2].

M[n]	Quotient	Remainder	Bit stream
0	0	0	0_00
1	0	1	0_01
2	0	2	0_10
3	0	3	0_11
4	1	0	10_00
5	1	1	10_01
6	1	2	10_10
7	1	3	10_11
8	2	0	110_00
9	2	1	110_01
10	2	2	110_10
11	2	3	110_11
12	3	0	1110_00
13	3	1	1110_01
14	3	2	1110_10
15	3	3	1110_11
16	4	0	11110_00

### 2.2. Algorithm

**Fig 1:**
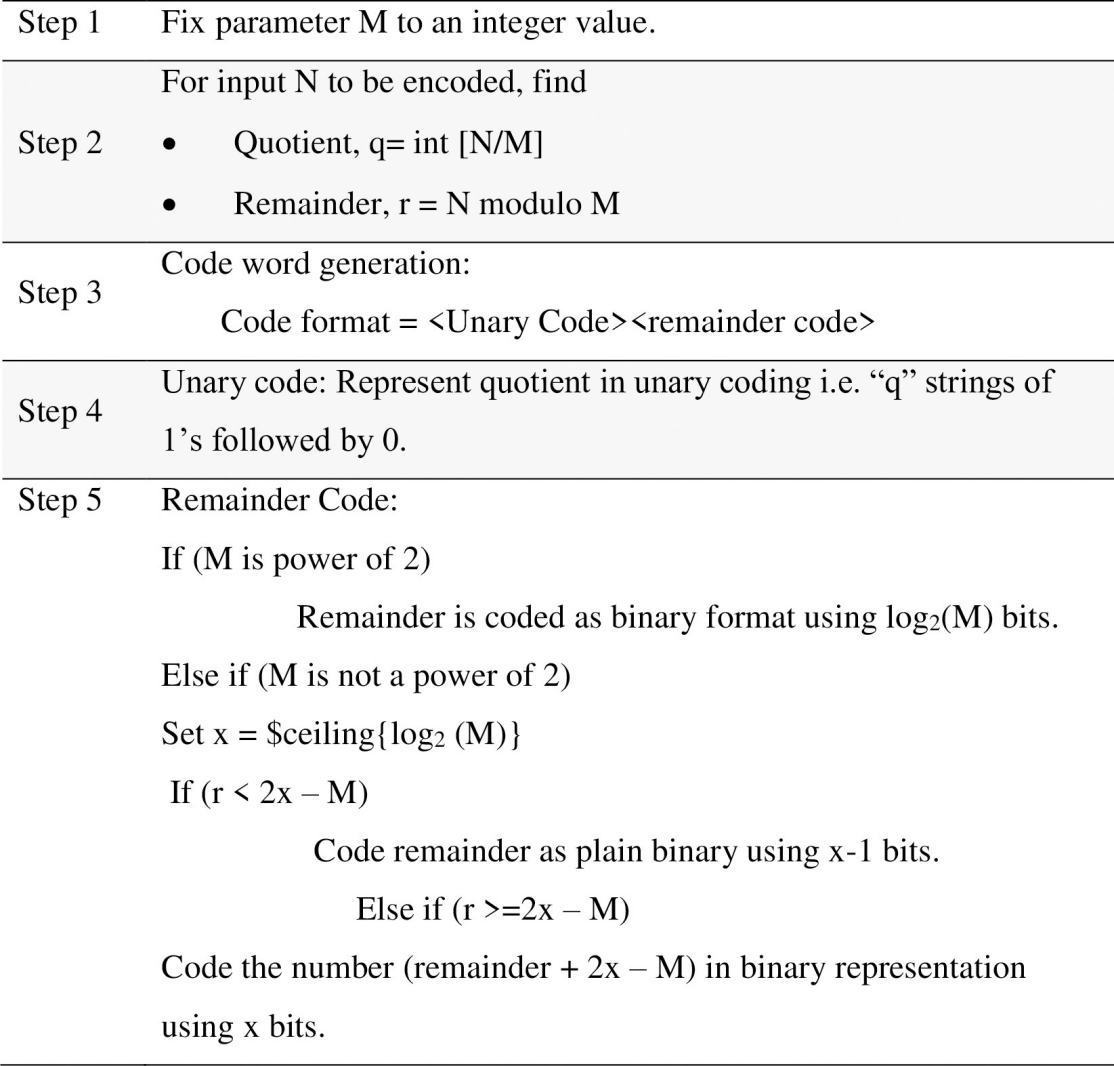
G R code algorithm.

The GR code algorithm is well explained by Fig 1. The code assigned for unary and Golomb code based on the value of M with appropriate bit assignment and the same is being explained below.

### 2.3. Technical gaps with the existing system architecture

**Fig 2:**
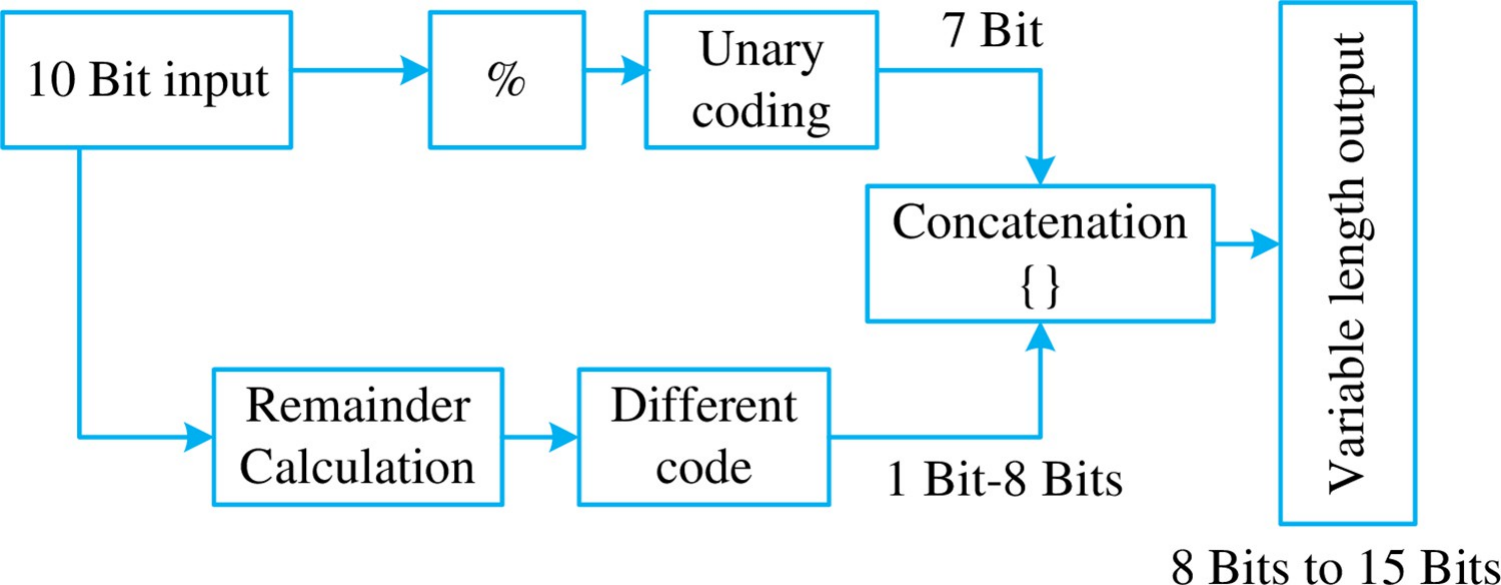
Block diagram for 10-bit input.

In Golomb-Rice code, the M -parameter greatly affects the encoding efficiency. In order to efficiently encode the data, the distribution of the data needs to be studied and accordingly the M—parameter needs to be selected. In this case, the value of M is assumed to be 128 and the input data to be encoded is assumed to be 10-bit in size. This setup enables the ‘q’ value to vary from as low as zero to as high as 7. The variable ‘q’ holding the value 7 means that the unary code will be 8 bits at its maximum length. This method does allow a certain compromise on data compression by decreasing the bit length of most used input symbols. However, it also increases the length of lesser-used symbols to a very large extent. This makes GR code unsuitable for use when the data is not completely geometrically distributed. This is well illustrated in Fig 2 with an input bit size of 10.

## 3. Proposed methodology

**Fig 3:**
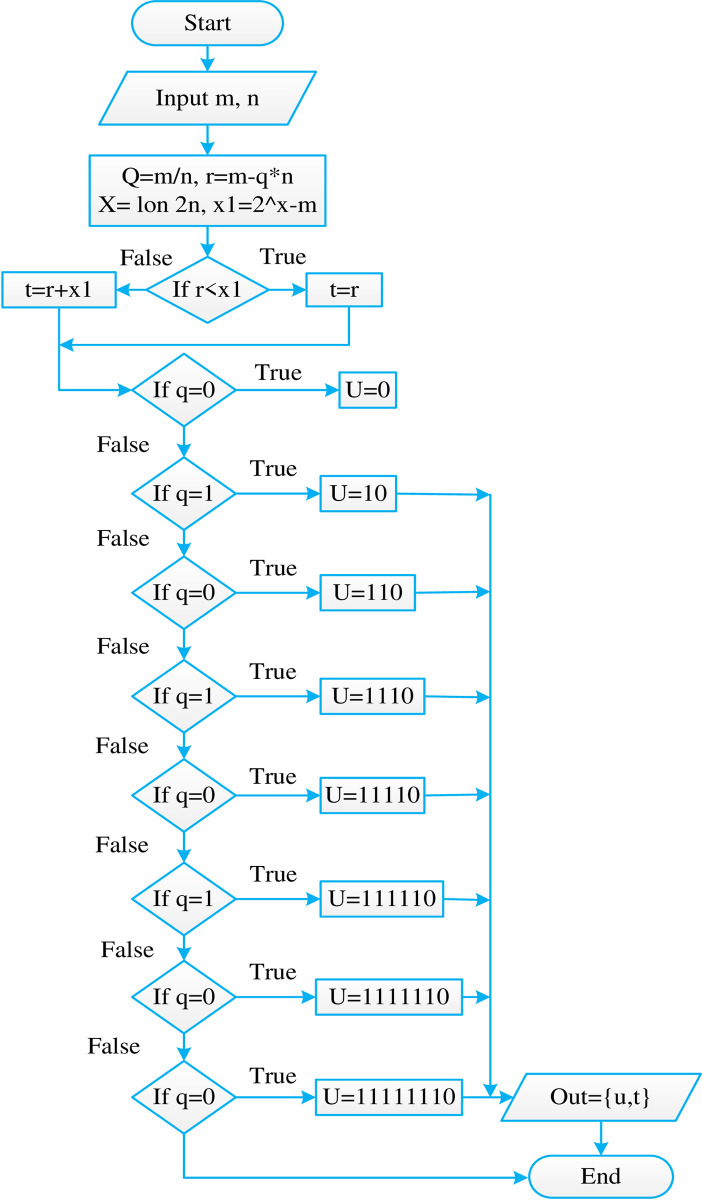
Flowchart for GR codes.

This work aims to reduce the bit length used for unary code. The selected value of M as 128 allows us an ideal case to show various types of methods to reduce the length of unary code while not being too large to handle in the scope of this paper. The implementation of a normal GR encoding scheme was carried out using Verilog coding. The outputs were observed and matched with expected results in order to verify whether the code worked with all possible 10-bit inputs. Further, an encoding method was applied which converted all the possible unary codes to fixed length 3 bit code. This helped in reducing the complexity of the code but the bit-length of the output was back to 10-bits for all the inputs, which meant there was no compression in the end. The input was 10 bits and the output was 10 bits as shown in Fig 3.

**Fig 4:**
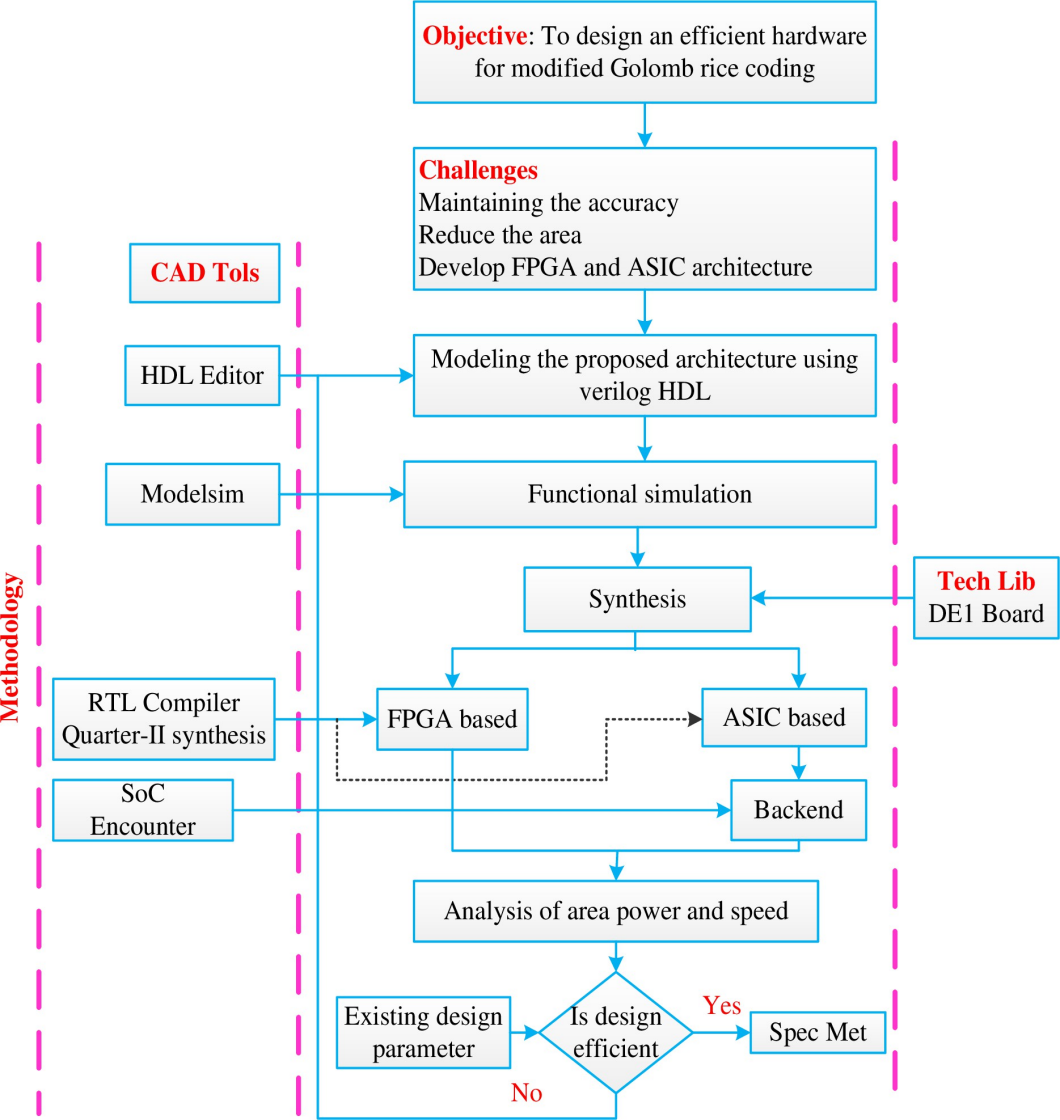
Design methodology followed for the hardware.

However, this allowed another modification where there was a separate encoding scheme for unary codes. Next, the algorithm was modelled using Verilog, simulated using modelsim and synthesized using Cadence Encounter RTL compiler. This meant including an entire division module and a loop to find the log values for different values of parameter M. These codes were then synthesized. Various reports like timing, area, delay and power were obtained for all proposed 3 schemes and the original GR encoding scheme as shown in Fig 4. Comparison of all the data was visualized using graphs. Final conclusion was drawn as to which method was better for which data distribution.

### 3.1. Proposed schemes

This scheme tries to reduce the three largest bit sized unary codes by encoding them using 2 bits. This allowed the unary part of the code to shorten its bit length. The codes which were 6,7,8 bit in size were 2 bit each now. The maximum possible bit length of the unary code was 5 bits now. This further led to another two schemes, which used encoding scheme to fix their lengths. In order to decode this code, the bit length needs to determined and accordingly further steps are taken. The possible lengths of unary codes are 1,2,3,4,5 bits. This means that the final combined GR code will be 8,9,10,11,12 bit long. This means whenever the codes are 8,10,11,12 bit long, normal GR decoding will take place which involves finding the leading 0. Whereas for 9 bit long codes the first 2 bits will always determine their unary codes and the remaining 7 bits will correspond to the respective remainder. Similarly for schemes 2 and 3, their unary codes were changed to reduce the bits used.

### 3.2. High speed Golomb Rice Code (HSCRC) proposed scheme-1

The unary part of the code is modified to decrease the amount of bits used. Here, we have taken the example of a 10-bit input. The value of M is 128. This gives us a q value ranging from [0,7]. The maximum bit size of output will be 15 bits [8 unary bits + 7 remainder bits]. In this, 7 remainder bits are definite in size and are not changed. The unary representation according to GR code will be as shown in Table 2.

**Table 2:** (HSCRC) encoding scheme.

Value of “q”	Unary representation	Further encoding	Bits saved
0	0	0	-2
1	10	1	-1
2	110	10	0
3	1110	11	1
4	11110	100	2
5	111110	101	3
6	1111110	110	4
7	11111110	111	5

Use of this encoding technique reduces the number of bits used but it also neutralizes the effect of GR coding. Since, the input was 10 bits in size and the output also remains 10 bit in size, there was no actual compression. This is nothing but just a direct binary representation of the input albeit in a different way.

**Fig 5:**
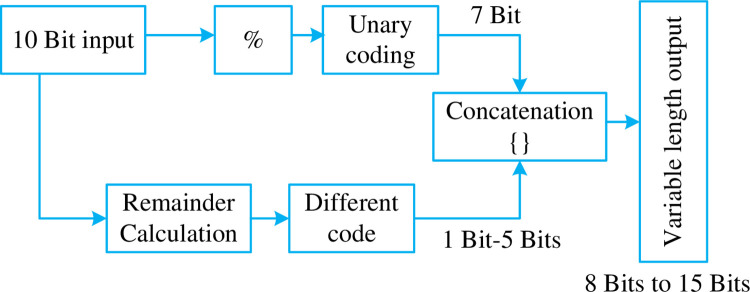
Block diagram for (HSCRC) Scheme-1 taking in a 10-bit input.

We needed to encode the input in such a way that the reduction in bit used is significant even in comparison to direct binary representation. Here, we propose a new method to encode the unary part. The last 3 unary codes are represented using two bits instead of their original codes in Table 3. As a result, the concatenated string will be smaller in size as compared to the original as shown in Fig 5. The GR coding can be of 15 bits out of which 7 bits are fixed for remainder and they can’t be altered, remaining 8 bits vary in unary code as the Q values changes from Q = (1–7) which takes 8 bits maximum for representing that in Unary code.

**Table 3:** (HSCRC) Scheme-1 encoding.

Value of “q”	Unary representation	Further encoding	Bits saved
0	0	0	0
1	10	10	0
2	110	110	0
3	1110	1110	0
4	11110	11110	0
5	111110	0	4
6	1111110	1	5
7	11111110	11	6

Let us consider a 10 bits input, the last 7 bits are fixed for remainder calculation. The first 3 bits are used for different Q values ranging from 1–7 values, this again gives us the same bit size as input which result in no compression so this work suggest an alternative idea to represent the Unary representation of Q value. In this method a maximum of 12 bits is required compared to 15 (i.e) 7 bits for differential code (for remainder value) and a maximum of only 5 bits for unary code there by saving 3 bits for representation.

### 3.3. Low Power Golomb Rice Code (LPCRC) proposed Scheme-2

Also, we can use the representation according to our needs as the data distribution differs. If the data distribution is linear, then the above method saves most bits. However, if the data distribution is geometric in nature the distribution below is better. The modified encoding scheme is shown in Table 4.

**Table 4:** LPCRC-Scheme- 2 encoding.

Value of “q”	Unary representation	Further encoding	Bits saved
0	0	0	0
1	10	10	0
2	110	0	1
3	1110	1	2
4	11110	11	3
5	111110	111110	0
6	1111110	1111110	0
7	11111110	11111110	0

**Fig 6:**
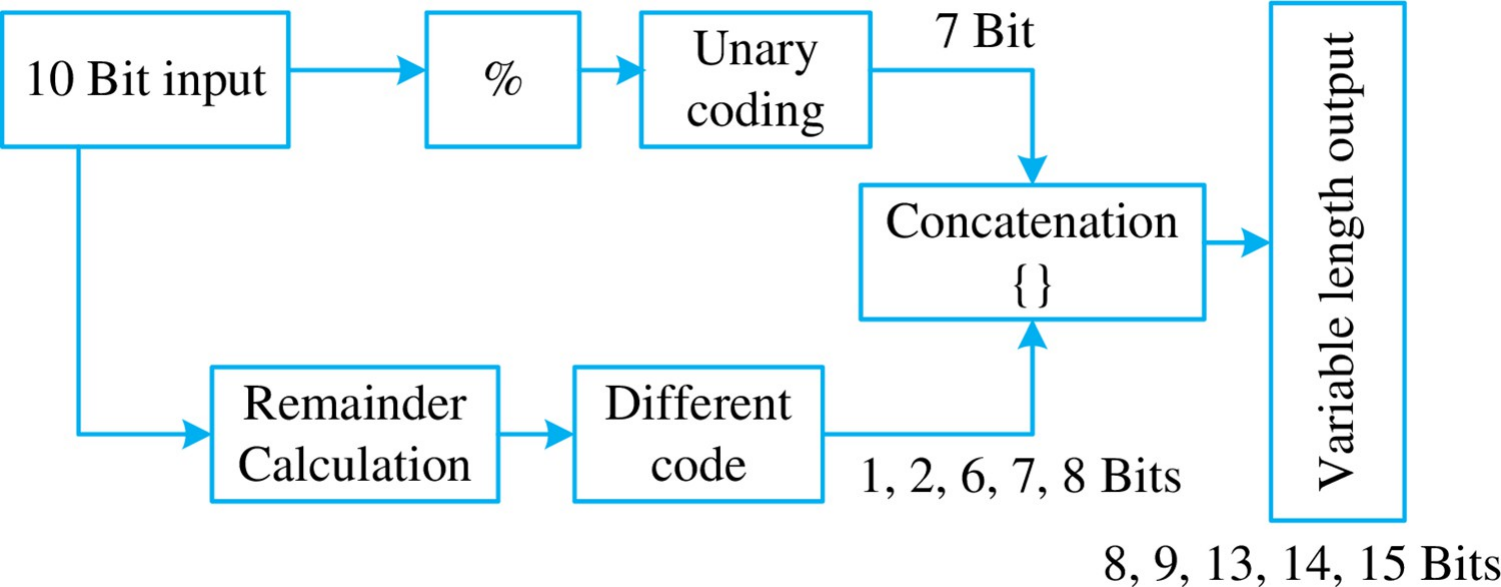
Block digram for LPCRC-Scheme- 2 taking in a 10-bit input.

As a result, the block digram of the encoding scheme changes and is shown in the Fig 6.

### 3.4. Efficient bit Reduction Golomb Code (EBRGC)-proposed Scheme-3

Further, we can make use of different sized registers by encoding the inputs even more judiciously. If the output registers are defined for 3 different bit sizes i.e. 8, 9, 10 bits, we can effectively use as shown in Table 5.

**Table 5:** (EBRGC)-Scheme- 3 encoding.

Value of “q”	Unary representation	Scheme-3 encoding	Bits saved
0	0	0	0
1	10	10	0
2	110	0	1
3	1110	1	2
4	11110	11	3
5	111110	0	3
6	1111110	1	4
7	11111110	10	5

**Fig 7:**
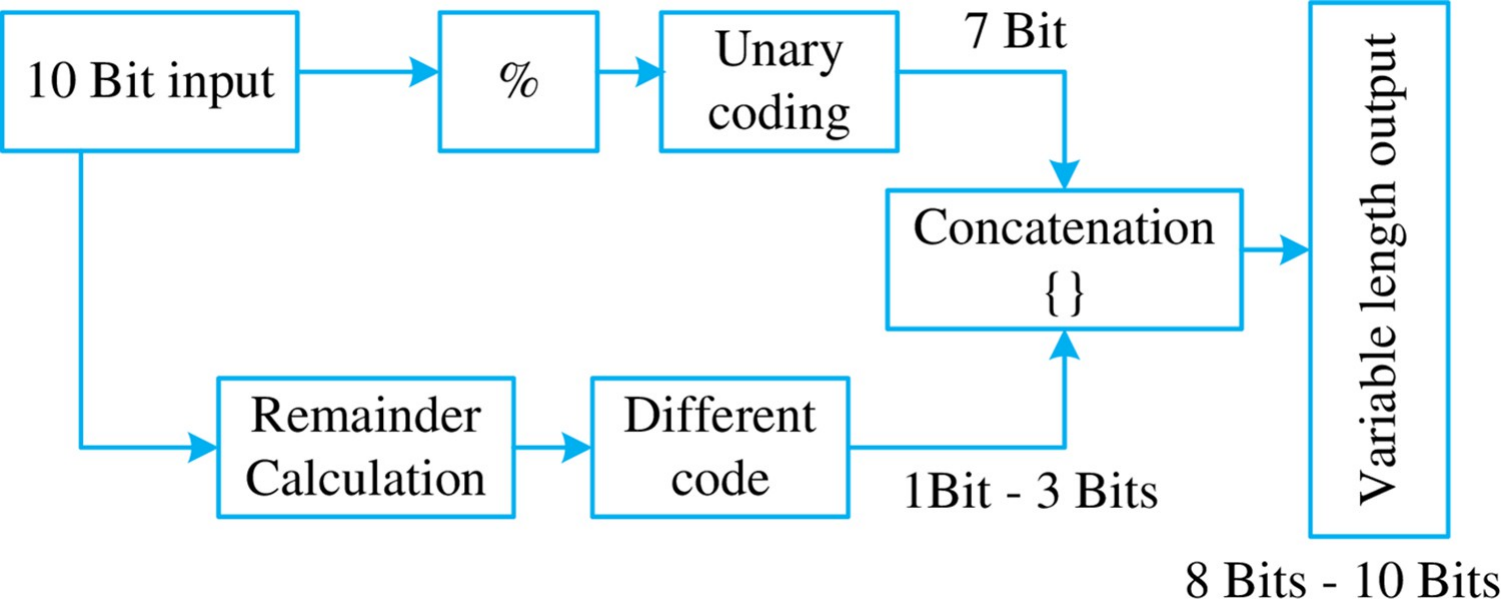
Block digram for (EBRGC) Scheme- 3 taking in a 10 bit input.

As a result, the block diagram of the encoding scheme changes and is shown in the Fig 7.

The output is taken through 5 registers of defined bit length. The registers are of 12,11,10,9,8 bits in size respectively. This makes sure that during decoding, it’s possible to decode the value without any error.

The proposed three schemes HSGRC (Scheme- 1) LPCGRC (Scheme- 2) EBRGRC (Scheme- 3) follows variable input and variable output coding schemes based on the metrics of optimization namely bit reduction, power or delay optimization. In this connect the unary code for the 6,7,8 may have same bit size but the ‘differential code’ part will have different value for remainder and here by it makes a difference in the code generation. This scheme of code generation at encoder will get reversed on the decoder side to maintain the validity of the algorithm.

## 4. Performance analysis

The proposed system is mathematically worked out to verify the validity of the changes which we have made and the same was designed for its hardware architecture. The hardware modelling was done using Verilog and the same was simulated using Modelsim. In order to get better optimization with design metrics like area, power and timing, the proposed design was synthesized using 45nm TSMC design lib with cadence RTL compiler. The synthesized results are compared with the existing architecture in terms of area, power and timing and the same has been shown in table and graphs.

### 4.1. Bit reduction

**Fig 8:**
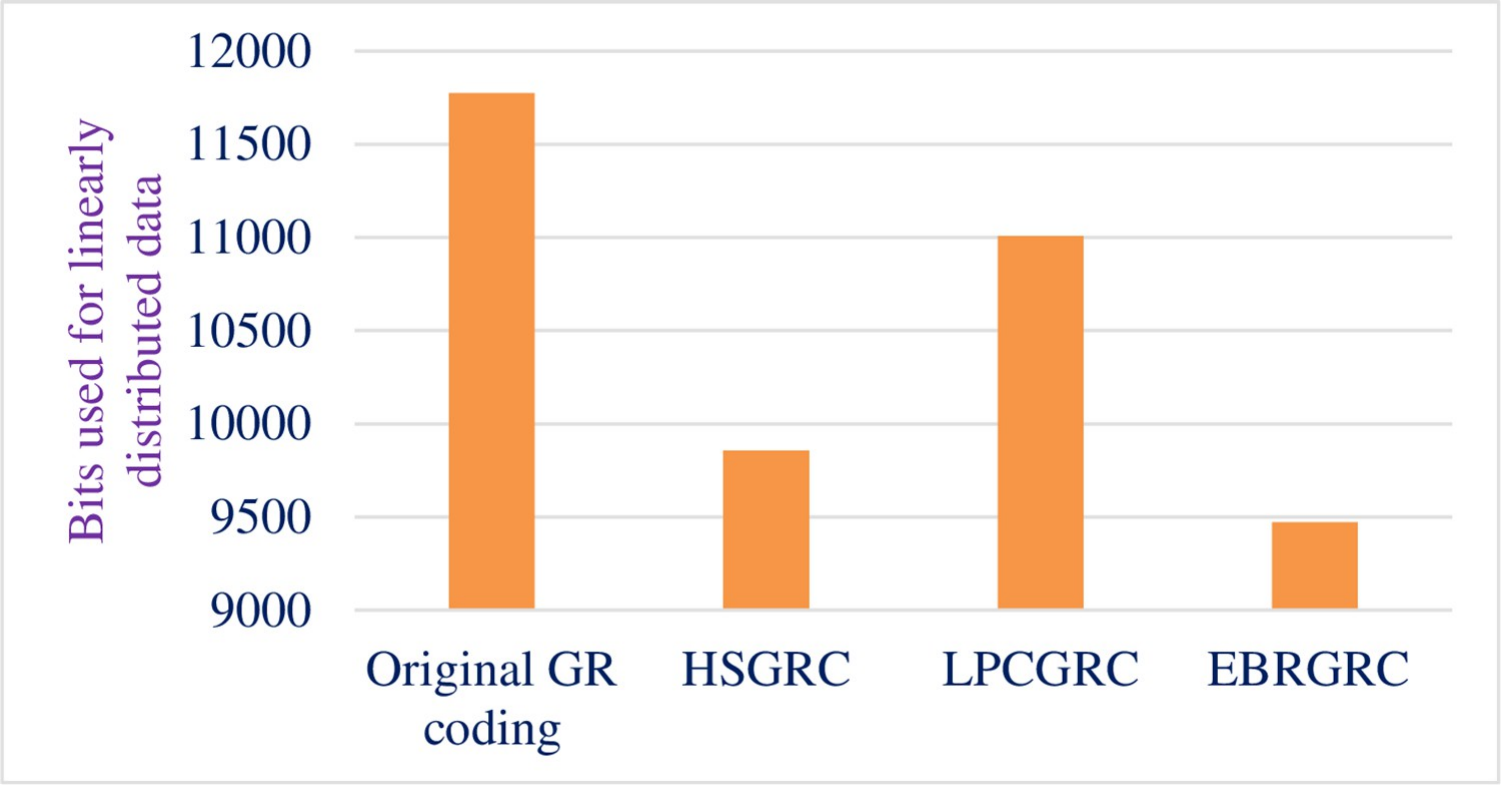
Comparative graph depicting bit usage.

This bit reduction is for linear test input. Total bits used were calculated while keeping in mind that every data has same occurring frequency and comes once. A total of 1024 different inputs are considered to fed into the system one by one. All the other methods use the same inputs but with a different way to encode unary part of the code. Table 6 shows the percentage of bit reduction for the three methods proposed. Fig 8 shows the graphical display of bit usage for the conventional and proposed GR coding techniques.

**Table 6:** Data showing number of bits used.

Method Used	Bits Used	Bits saved	% Reduction
Original GR coding	11776	-	-
HSGRC (Scheme- 1)	9856	1920	16.30%
LPCGRC (Scheme- 2)	11008	768	6.52%
EBRGRC (Scheme- 3)	9472	2304	19.56%

### 4.2. Timing report

**Fig 9:**
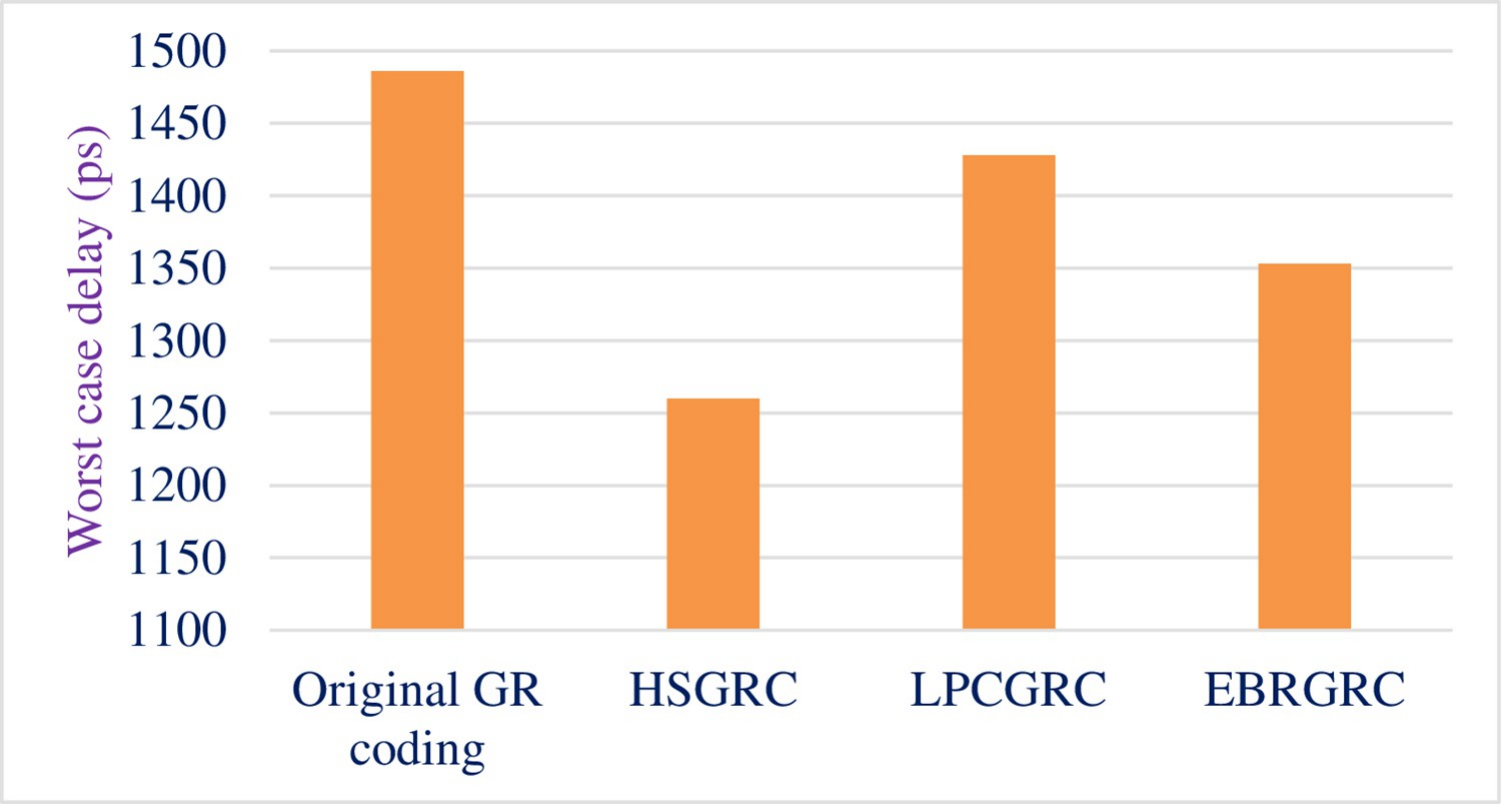
Comparative graph depicting worst case delay.

The synthesized report gives the shows the worst case delay for original algorithm and the proposed methods. Table 7 compares the worst case timing for all 3 modified ways of encoding the same input data. Fig 9 shows the graphical representation of the same.

**Table 7:** Data showing change in worst case delay.

Method Used	Worst Case Time Delay (ps)	Time reduction (ps)	% Reduction
Original GR coding	1486	-	-
(HSGRC)Scheme- 1	1260	151	10.16%
(LPCGRC)Scheme- 2	1428	58	3.90%
(EBRGRC)Scheme- 3	1353	133	8.95%

#### 4.3. Area report

**Fig 10:**
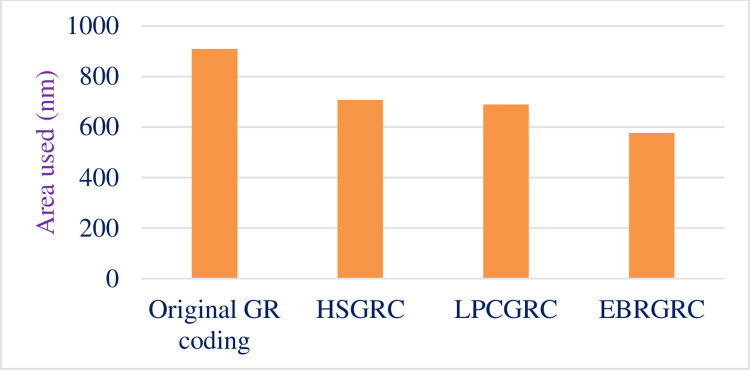
Comparative graph depicting area used.

Area analysis is being made on the area report obtained on the 90nm TSMC synthesis. Table 8 shows the total area used for synthesizing the original algorithm and compares the data for all 3 modified ways of encoding the same input data. Fig 10 shows the graphical representation of the same.

**Table 8:** Data showing change in area.

Method Used	Area(nm)	Area reduction (nm)	% Reduction
Original GR coding	909.2	-	-
HSGRC (Scheme- 1)	707.2	202	22.21%
LPCGRC (Scheme- 2)	689	220.2	24.21%
EBRGRC (Scheme- 3)	577	332.2	36.53%

### 4.4. Power report

**Fig 11:**
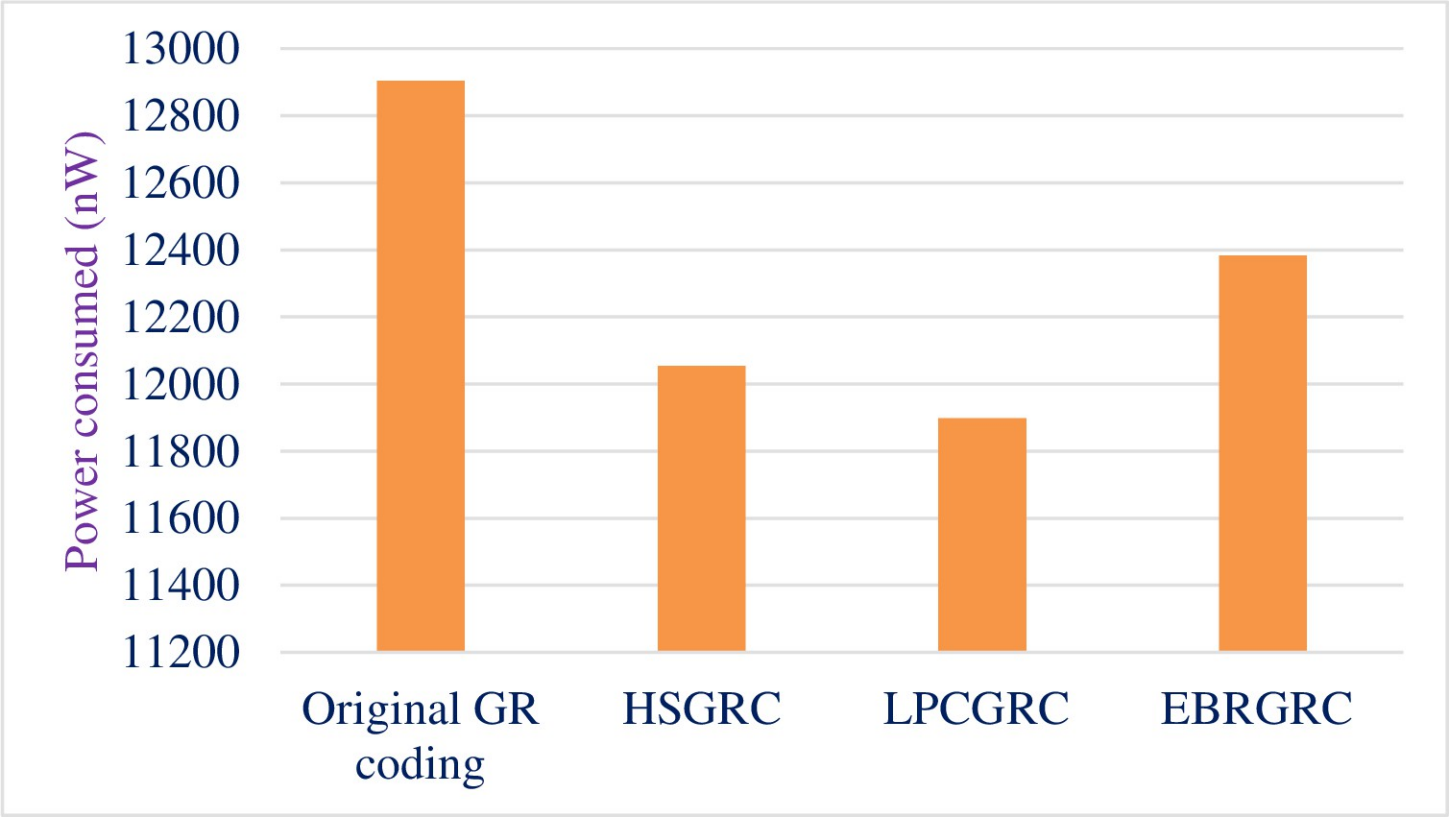
Comparative graph depicting power consumption.

**Fig 12:**
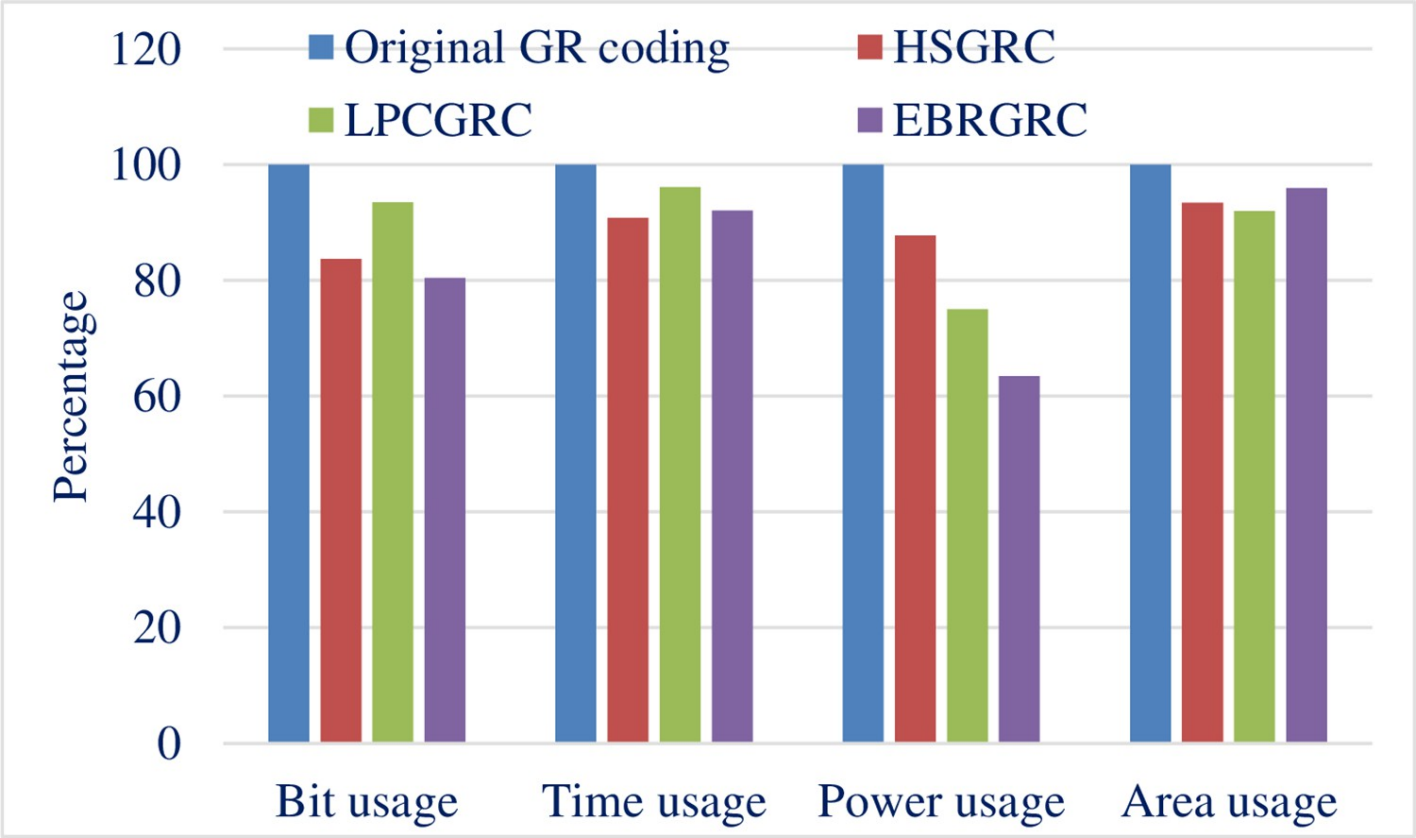
Comparative bar graph depicting difference between the methods.

Table 9 shows the total power consumed by the proposed circuits made with the original algorithm and compares the data for all 3 modified ways of encoding the same input data. Fig 11 shows the graphical representation of the same. Fig 12 shows a comparative bar graph diagram for the proposed methods.

**Table 9:** Data showing reduction in power usage.

Method Used	Power Consumed (nW)	Power saved(nW)	% Reduction
Original GR coding	12904.65	-	-
HSGRC (Scheme- 1)	12054	850.65	6.59%
LPCGRC (Scheme- 2)	11899	1005.65	7.79%
EBRGRC (Scheme- 3)	12838.5	521.15	4.03%

**Fig 13:**
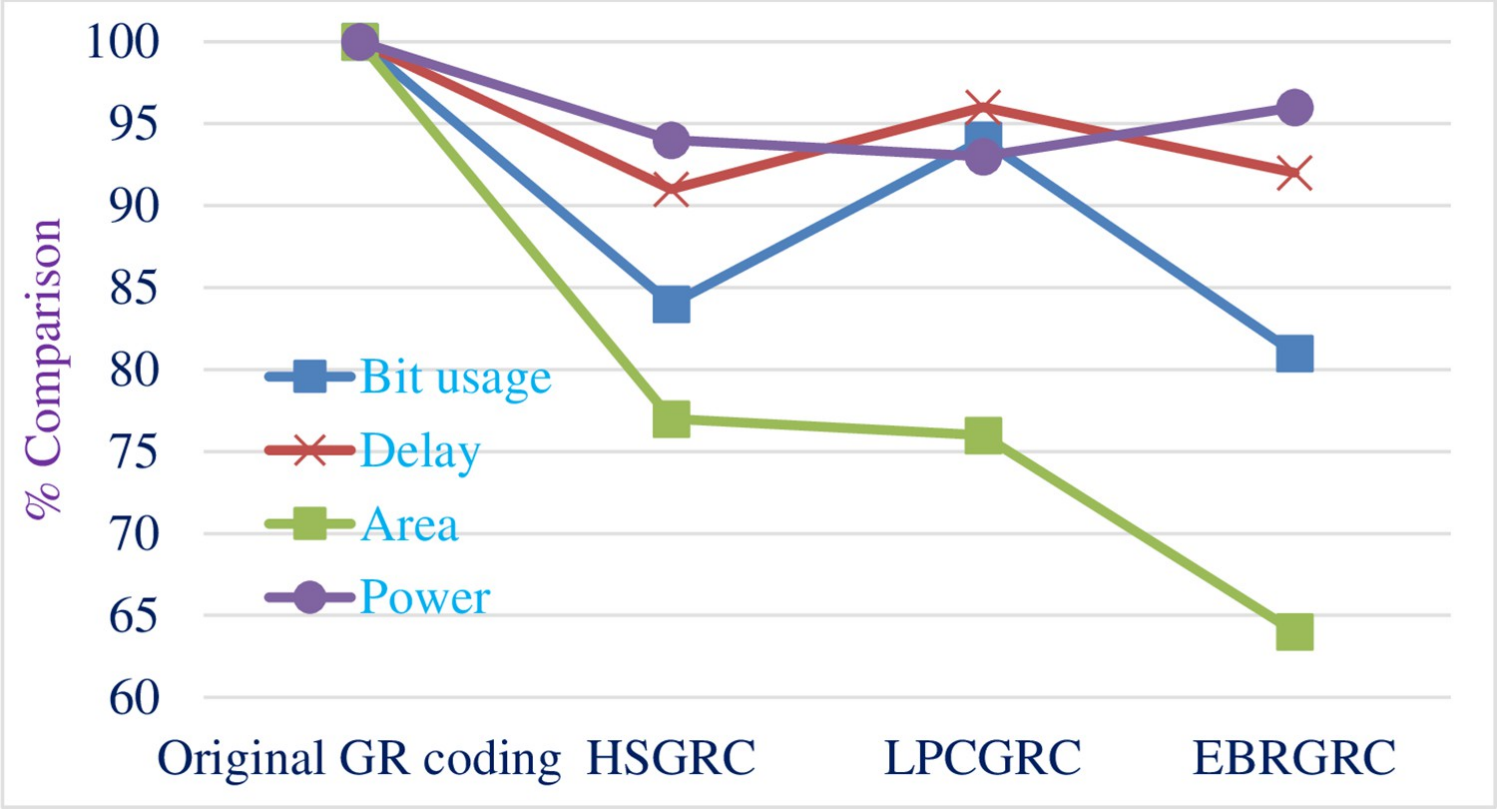
Comparative line graph depicting variations in the proposed methods.

**Fig 14:**
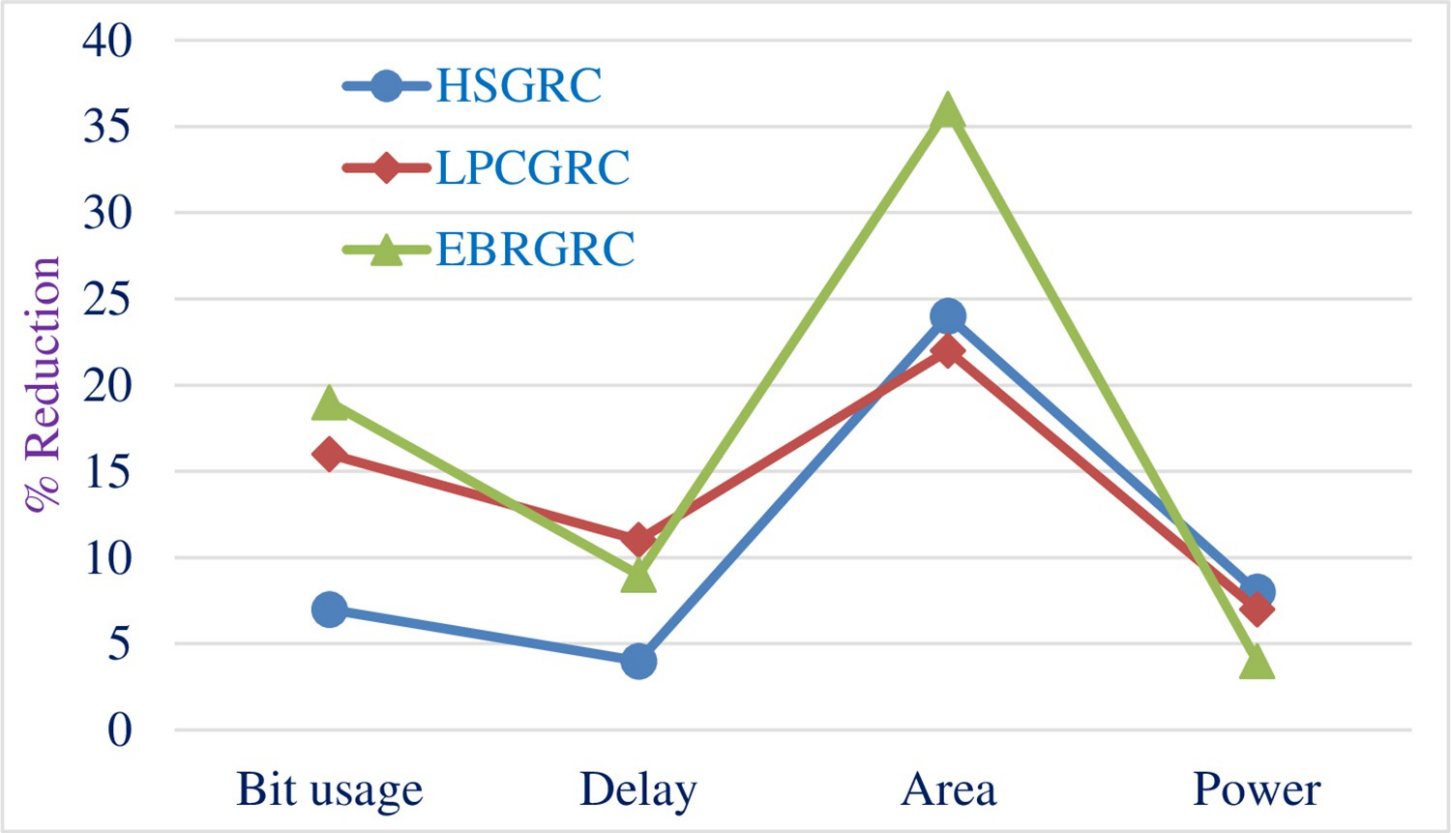
Comparative line graph depicting improvements in proposed parameters.

Then, we have a different way altogether to show this data where we compare the relative improvement in terms of percentage. Figs 13 and 14 shows a line graph comparison of different methods and metrics used respectively. This is a relative comparison because of which all the readings are compared relatively to each other on ascale of 100 where 100% is the reading for original GR encoding algorithm.

**Fig 15:**
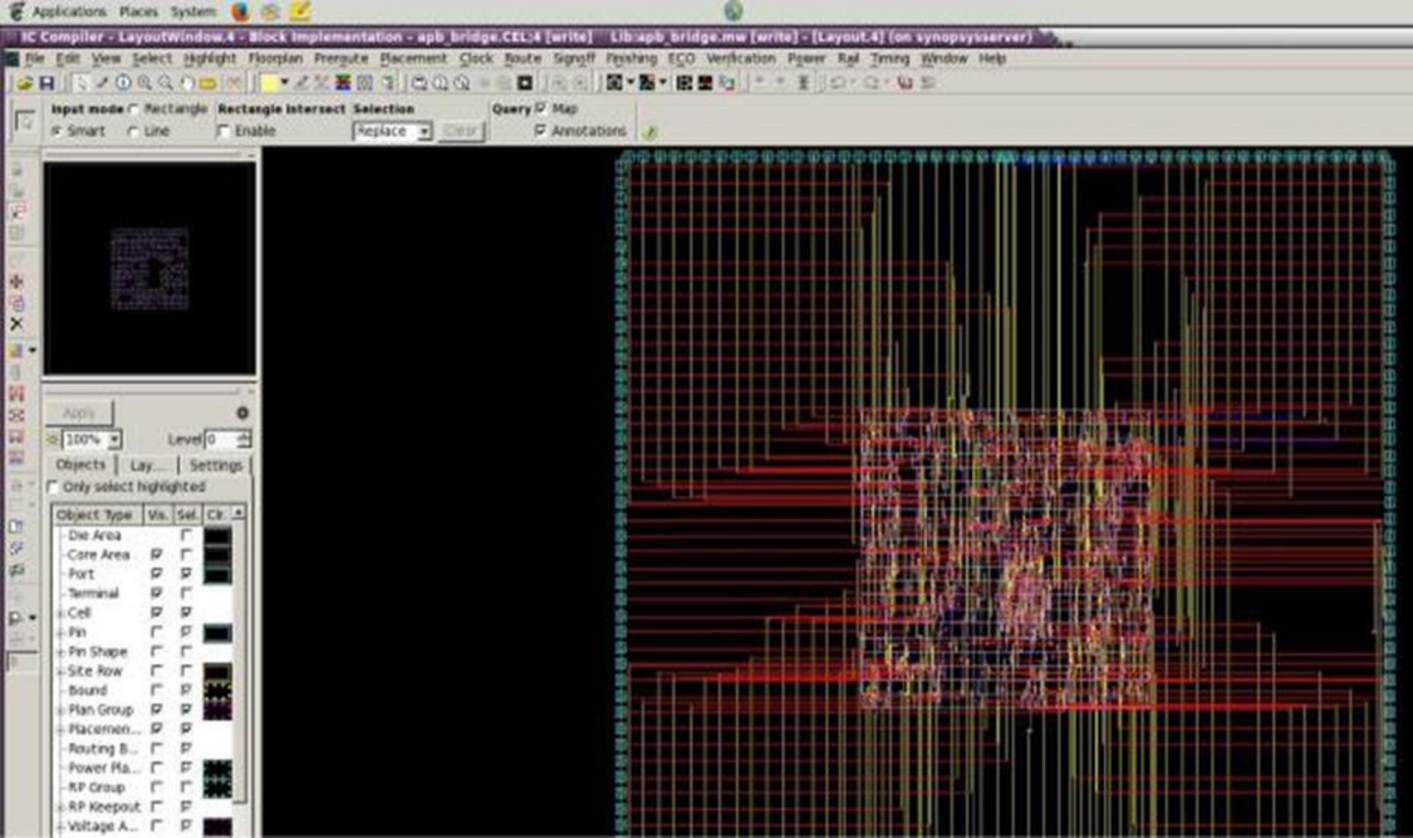
Complete layout of the proposed architecture.

The readings for original GR encoding algorithm is considered as the benchmark and hence it is shown as 100% while others are compared relatively to the 100% value. To benchmark our results with the existing state of art design we made a comparative analysis with those existing hardware design for data compression. The comparison is made in the aspect of hardware metrics like area, power, frequency of operation with the original GR coding and our proposed designs and also with state of art design with other researchers. Table 10 shows the comparative results. The proposed design were coded in Verilog HDL and the RTL functional verification is carried out using synopsis VCS tool followed by synopsis Design Compiler for synthesis and the complete backend like partitioning, placement, routing and clock tree synthesis were carried out using synopsis ICC tool. Fig 15 shows the optimized synthesized and routed proposed design with all design optimization being implemented.

**Table 10:** Comparative results with state of art design.

Method Used	Area	Power consumed (nW)	Bits used	Speed (MHz)
Original GR coding	909.2 nm^2^	12904.65	11776	672
HSGRC (Scheme -1)	707.2 nm^2^	12054	9856	793
LPCGRC (Scheme -2)	689 nm^2^	11899	11008	700
EBRGRC (Scheme -3)	577 nm^2^	12838.5	9472	739
Wei Jhih et al.[33]	6.26mm^2^	405900000	*	255
Xiaoke Qin [34]	250 slices	*	*	195
Hang wang et al.[35]	13.5 Logic gates	*	*	600

## 5. Application and future scope

The synthesized results of the proposed design shows an appreciable improvement of around 15% reduction in bit size, around 25% reduction in area, 6% reduction in power consumption and 9% increase in speed. Since there is a reasonable amount of bit reduction it finds a great application in test vector compression and response compaction of VLSI testing [27–30,36–51]. It immensely reduces the testing power because the switching power get reduced due to the bit reduction followed for coding [32,52]. The wide application of AI and IoT requires a huge data manipulation and storage which essentially demands an effective hardware architecture for lossless data compression coding and decoding [34,35,53,54].

Biomedical applications and neuromorphic computing require huge data computation and lossless compression for analysis, prediction and classification. This majorly demands a dedicated efficient hardware’s for data compression [46–51].

This work can be further extended by applying signal statistics based modified GR codes architecture for Machine learning algorithms and this could also be optimized based on stochastic computing based architecture which could be applied in robotics and brain computing.

The most advantages of GR coding implementations are its very high compression ratio, analytically predictable compression results, and a low-cost and scalable on-chip decoder. Golomb-Rice code is reversible and error-detectable so the code is more robust and efficient in transmission and decompression which is used in many medical image processing. Even though GR offers high compression ratios, there exists a gap in the performance of the decompression architecture because it operates through bit-serial processing but there are many reported works added in [55–58] which come up with parallel efficient architectures for test vector compression and decompression. This work could be extended for building an efficient hardware architectures for decoding algorithms.

The Golomb-Rice (GR) method follows bit-wise techniques for data compression which provides better compression ratio and it is applied to the compression of images, audio files and lists of inverted indices etc. However, since GR is a serial algorithm, decompression is regarded as a very slow process but efficient parallel hardware were developed to increase its performance. Works [33,59,60] shows a no-stall hardware architecture, capable of decompressing streams of integers compressed using the GR method, at a rate of several bytes (multiple integers) per hardware cycle. All these work suggest the use of GR technique for data stream.

This work can also be extended for efficient use of decompression GR architectures for exponentially distributed test data [33,59,60].

**Fig 16:**
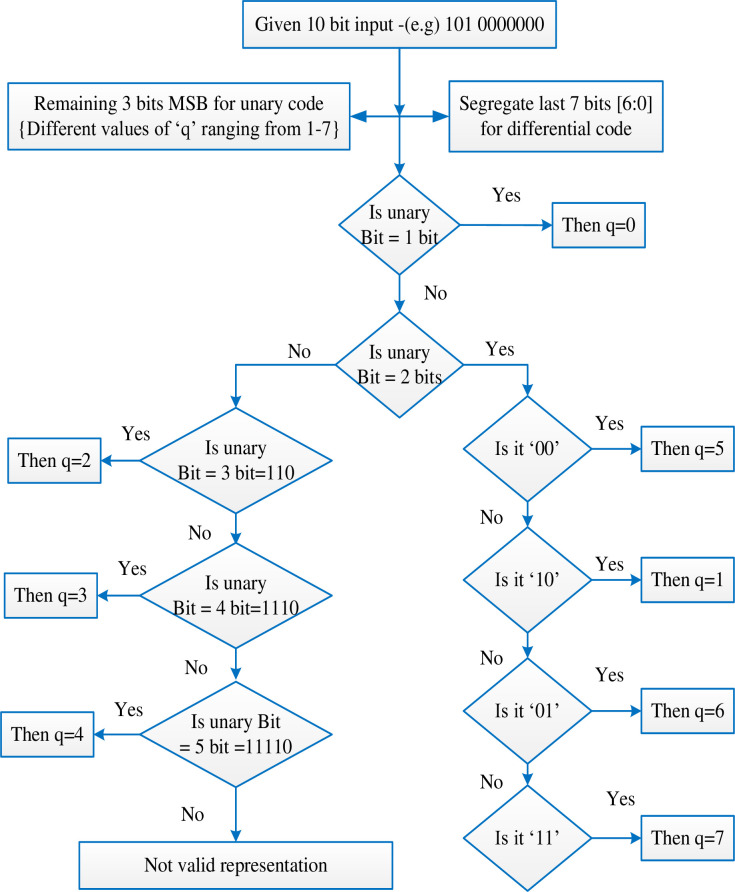
Decompression algorithm for the proposed HSCRC encoding.

To understand the decompression, let us consider a 10 bits input, the last 7 bits are fixed for remainder calculation. For this scenario we have 3 bits for unary representation in the proposed method this 101 is not a valid unary representation so for the value of q = 5 we represent them with ‘00’. Since the encoding method follows the Table 3 the decoding also follows the same as shown in Fig 16. The proposed decoding for the given 10 bit input is as follows in the flow chart.

## 6. Conclusion

This work tried to come up with three different algorithmic level modification in GR coding and its corresponding hardware architectures were designed, simulated and synthesized. The synthesized results were compared which shows an effective increase of optimal design metrics compared with GR compression algorithm. These modifications offered significant improvements. These improvements have been quantified and summed up below:

**HSGRC (Scheme -1)** The synthesized results display a 16.30% reduction in bit usage for linearly spread data. Worst-case delay had been reduced by 10.16%, while area had been reduced by 22.21%. In addition, power consumption is down by 6.59%.

**LPCGRC (Scheme -2)** The synthesized results demonstrates 6.52% reduction in bit usage for linearly spread data. Worst-case delay had been reduced by 3.9%, while area had been reduced by 24.21%. In addition, power consumption is down by 7.79%.

**EBRGRC (Scheme -3)** The synthesized results shows 19.56% reduction in bit usage for linearly spread data. Worst-case delay had been reduced by 8.95%, while area had been reduced by 36.53%. In addition, power consumption is down by 4.03%.

Based on the comparison of results with the existing architecture the results suggest for the usage in test vector compression for VLSI testing environment, biomedical image compression and transmission, data transmission from edge to cloud and viceversa in AI with IoT applications.

## Supporting information

S1 File The code used in Fig 15 is uploaded in supporting files. (PDF)
