# Supplementary material for: Hardware optimization for effective switching power reduction during data compression in GOLOMB rice coding
Source: PLoS One. 2024 Sep 26;19(9):e0308796. doi: 10.1371/journal.pone.0308796 (PMC11426500; doi:10.1371/journal.pone.0308796)
Supplement: S1 File — (PDF) [file pone.0308796.s001.pdf]

```
import math

# RICE ENCODER
def rice_encoder(S, M):
     # If sample value is negative, consider it positive
     # and store signs separately
    if S < 0:
        S = -S
        sign = "-"
    else:
        sign = "+"

    # Compute K using equation, M = 2^K
    K = int(math.ceil(math.log(M, 2)))

    # For S, the number to be encoded, find q and r
    quotient = int(math.floor(S / M))
    remainder = S % M

    # Generate codeword
    q_code_str = ""
    for i in range(quotient):
        # Add 1 q number of times.
        q_code_str += "1"

    # Find the difference
    diff = int(math.pow(2, K) - M)

    # Write out r in binary
    if (remainder < diff):
      b = K - 1
      a = "{0:0" + str(b) + "b}"
      r_code_str = a.format(remainder)
    else:
      b = K
      a = "{0:0" + str(b) + "b}"
      r_code_str = a.format(remainder + diff)

    # Encoded string
    code_word = sign + q_code_str + "0" + str(r_code_str)

    return code_word

# RICE DECODER
def rice_decoder(S, M):
    # Separate the sign and K-bits binary
    sign = S[0]
    S = S[1:]

    # Compute K using equation, M = 2^K
    K = int(math.ceil(math.log(M, 2)))

    # Separate q and r by spliting at the first 0
    q_and_r_list = S.split('0', 1)

    # Get quotient, q, by counting number of 1s before the first 0
    q = len(q_and_r_list[0])
```

```
# Get remainder, r, by converting the next K-bits binary to decimal
r = int(q_and_r_list[1][:K], 2)

# Compute encoded number using q * M + r
S = q * M + r

# Change sign of the decoded bit
if sign == '-':
    S = -S

return S
```
